# Chemically reactive bioconvection flow of tangent hyperbolic nanoliquid with gyrotactic microorganisms and nonlinear thermal radiation

**DOI:** 10.1016/j.heliyon.2019.e03117

**Published:** 2019-12-30

**Authors:** Kamel Al-Khaled, Sami Ullah Khan, Ilyas Khan

**Affiliations:** a Department of Mathematics & Statistics, Jordan University of Science and Technology, P.O. Box 3030, Irbid, 22110, Jordan; b Department of Mathematics, COMSATS University Islamabad, Sahiwal, 57000, Pakistan; c College of Engineering, Majmaah University, P.O. Box 66, Majmaah, Saudi Arabia

**Keywords:** Computational mathematics, Industrial engineering, Thermodynamics, Theoretical fluid dynamics, Physics methods, Tangent hyperbolic nanofluid, Motile organisms, Variable thermal conductivity, Oscillatory stretching sheet

## Abstract

On the account of motivating fabrication of bioconvection phenomenon in various engineering and industrial systems, an attention has been devoted by researchers in current decade. Therefore, this theoretical investigation deals with the utilization of bioconvection phenomenon in flow of tangent hyperbolic nanofluid over an accelerated moving surface. It is assumed that the flow is generated due to periodically motion of the sheet. The energy equation is modified by entertaining the nonlinear thermal radiation features. The chemical reaction effects are elaborated in the concentration equation. Moreover, the significance of present flow problem increases by utilizing the thermophoresis and Brownian motion effects. The governing equations are transmuted into non-dimensional form with utilization of appropriate quantities. The analytical solution is computed by using homotopy analysis method. The implications of promising parameters on velocity profile, temperature profile, nanoparticles volume fraction and microorganisms profile is evaluated graphically. The presence of radiation parameter, thermophoresis and Brownian motion effects are more frequent for enhancement of heat transfer. The reported observations can efficiently use in the improvement of heat transfer devices as well as microbial fuel cells.

## 1 Introduction

The interest has been developed in recent few years towards the study of non-Newtonian materials by numerous instigators due to their valuable consequences and practical industrial, chemical, biochemical engineering and mechanical applications [1, 2, 3]. Accompanying some prestigious applications include blood, honey, cosmetics, glue, crude oil, asphalts, cream etc. Due to their complex molecular structure, the non-Newtonian fluids accomplish a nonlinear relationship with shear stress and rate of deformation and subsequently assigned in the category of power law model. In contrast to the viscous fluids, such fluids are more complex and the physical roperties of such fluids cannot be predicted by using simple relations. In order to overcome this issue, the scientists have suggested a variety of mathematical models regarding non-Newtonian materials in the literature. Among these mathematical models, the tangent hyperbolic model is one which accomplished the shear thinning effects i.e., the viscosity declined by increasing shear rate. Due to such interesting rheological behavior, many authors have used tangent hyperbolic model with distinct flow features. For instance, Nadeem et al. [4] constituted famous boundary layer approximation equations for the flow of tangent hyperbolic fluid past over a stretching surface. Hayat et al. [5] studied a two-dimensional flow of tangent hyperbolic fluid in presence of mass flux conditions and thermophoresis effects. Ullah and Zaman [6] directed the slip flow of tangent hyperbolic liquid over a stretched configuration. The dimensionless analysis for the governing equations has been performed via Lie group technique which was further tacked numerically with utilization of shooting procedure. The flow of tangent hyperbolic liquid in double saturated flow over a stretching cylinder has been signified by Nagendramma and co-workers [7]. Kumar et al. [8] numerically evaluated the involvement of heat absorption and generation features in tangent nanoparticles flow over a convectively heat surface. The numerical solution of the simulated flow problem was suggested by using shooting technique. Another interesting analytical based approach regarding flow of tangent hyperbolic nanofluid over an oscillatory moving surface was elaborated by Khan et al. [9]. Rehman et al. [10] discovered some interesting thermo-physical properties of tangent hyperbolic fluid over a confined regime. The peristaltic flow with variable thermal conductivity in tangent hyperbolic fluid was evaluated by Hayat et al. [11].

The transport of heat incorporating the fluid flow is necessitated in large number of nuclear and thermal-hydraulic processes. In order to enhance the heat transportation process, a variety of fluids and operating conditions has been tested. The interaction of such fluid into existing system may be useful in reduction of capital costs, improve the working efficiency and better design of desired system. Among the traditional methods, air is one of the primary methods to cooling the various electronic systems. However, it is often noted that for extremely higher heat fluxes, the role of liquid cooling is more progressive. The role of cooling is quite indispensable in order to sustain the desired thermal performances in various engineering and technological products like computers, motor engines, chemical reactions, laptops and cooling of strips. Nanofluids, with excellent thermo-physical features and relatively slow thermal resistance, are attributed as most attractive attention recently. Recently, the nanotechnology has been considered as most intriguing developments with effective cost and ultra-high output. The nanoparticles are relatively small sized particles and suspended in the liquid. The basic principle and innovation of such suspended particles is to enhance the thermal conductivity of widely used base liquids. The fundamental development on this topic was presented by Choi [12] which was further massively extended by various authors along with addition of some other physical features. For instance, Bhatti and Rashidi [13] observed the Brownian motion and thermodiffusion features in the flow of Williamson nanofluid plunged in porous medium. Hayat et al. [14] provided a mathematical model for melting heat with thermal radiation effects in stagnation-point flow of carbon nanotubes. Kumar et al. [15] exploited the cooling procedure based on involvement of Cuo-water based nanoparticles in the semiconductors. Ijaz et al. [16] inspected the entropy generation and activation energy phenomenon in flow of Sisko nanofluid along with interaction of nonlinear thermal radiation. Khan and Shehzad [17] analyzed the third grade fluid over periodically accelerated surface in addition of joule heating effects.

Microorganisms are unicellular organisms, they live everywhere as in animals, people and in bodies of plants. Microorganisms become cause of bioconvection as they are much thicker than water because of microorganisms gathering. Bioconvection phenomenon is presented by oxytactic bacteria that are up swimming micro-organisms. In fact, the bioconvection model is concerned with oriented swimming cell which is related with the microorganisms species. The physical interesting significance of bioconvection is successfully assorted in the bio-fuels, ethanol and in diverse industrial and environmental systems. Beside this, the bioconvection of nanoparticles is associated with the density of stratification along with the pattern formation which occurs due to interaction of microorganisms, buoyancy forces and nanoparticles. It is often observed that suspension stability of nanoparticles has been effectively enhanced in presence of gyrotactic microorganisms. This interesting phenomenon has been reported by many investigators in recent years. Shen et al. [18] described the velocity slip and jump boundary conditions in bioconvection flow of nanofluid in presence of thermal radiation. The thermophysical properties of nanofluids containing gyrotactic microorganisms with uniform free stream were numerically studied by Xu and Pop [19]. The study of water-based nanoliquid containing gyrotactic microorganisms over convectively heated surface has been directed by Khan et al. [20]. The stability analysis for gyrotactic microorganisms was performed by Saini and Sharma [21]. The features of bioconvection with the supremacy of gyrotactic microorganisms were encountered by Chakraborty et al. [22]. The bioconvection flow of nanofluid truncated in cone was investigated by Khan et al. [23]. Kumar et al. [24] investigated an unsteady stagnation point flow of bioconvective nanofluid with influence of thermophoresis, Brownian diffusion and slip velocity numerically by using Keller-box scheme. Mabdood et al. [25] determined the analytical expressions for free convective non-Newtonian flow with gyrotactic microorganisms. Khan et al. [26] investigation the slip flow of nanofluid with gyrotactic microorganisms over vertical plate. In another contribution, Khan et al. [27] analyzed the bioconvection in non-Newtonian fluid over a vertical surface in presence of free stream velocity.

Acutely observing the above highlighted review, it is noted that the bioconvection effects in flow of tangent hyperbolic nanoparticles in presence of gyrotactic microorganisms and nonlinear thermal radiation has not been presented yet. Unlike to the typical analysis, here flow is induced by periodically accelerated and moving surface. It is carefully examined that such features are not investigated and consequently aim is to full fill this gap. Fluid flow over accelerated moving configuration proved a seriousness application even in recent days due to industrial and physical applications like polymer processing, petrochemical industry, manufacturing processes, various geophysical systems, glass production, hot rolling etc. The current analysis has been performed by simulating nonlinear thermal radiation features which encountered wide range of applications in thermal extrusion phenomenon, solar system, missile technology, environmental applications, heavy mechanical apparatus, fission and fusion reactions and many more. The solution of inserted problem is carefully computed via homotopy analysis scheme.

## 2 Problem formulation

In current analysis, the tangent hyperbolic nanoliquid flow over an accelerated moving surface has been analyzed. The flow is assumed to be unsteady and incompressible. The accelerated configuration is periodically moving with velocity $b\bar{x}\sin\;\omega t$, where ω is being frequency. In cartesian coordinates, u is assigned in $\bar{x}$ direction while v is determined in $\bar{y}$ directions. The temperature, concentration and motile organisms near the surface is notified by $T_{w},C_{w}$ and $n_{w}$, respectively. The thermal radiation features are captured with help of Rosseland approximation. Further, the chemical reactions are also in included in the concentration equation. The flow equations for the tangent hyperbolic nanofluid based on such assumptions in addition of motile microorganism are represented in following forms:

(1) $$\frac{\partial u}{\partial \bar{x}}+\frac{\partial v}{\partial \bar{y}}=0,$$

(2) $$\begin{array}{l} \frac{\partial u}{\partial t}+u\frac{\partial u}{\partial \bar{x}}+v\frac{\partial u}{\partial \bar{y}}=\nu \;\frac{\partial^{2}u}{\partial {\bar{y}}^{2}}+\sqrt{2}\Gamma \nu \frac{\partial u}{\partial \bar{y}}\frac{\partial^{2}u}{\partial {\bar{y}}^{2}}\left(-\frac{\sigma^{*}B_{0}^{2}}{\rho_{f}}+\frac{\nu \varphi }{k^{*}}\right)u,\\ +\frac{1}{\rho_{f}}\left[\left(1-C_{\infty }\right)\rho_{f}\beta^{*}g\left(T-T_{\infty }\right)-\left(\rho_{p}-\rho_{f}\right)g\left(C-C_{\infty }\right)-\left(n-n_{\infty }\right)g\gamma^{*}\left(\rho_{m}-\rho_{f}\right)\right],\; \end{array}$$

(3) $$\frac{\partial T}{\partial t}+u\frac{\partial T}{\partial \bar{x}}+v\frac{\partial T}{\partial \bar{y}}=\left(\alpha +\frac{16\sigma_{m}T_{\infty }^{3}}{3k^{*}{\left(\rho c\right)}_{f}}\right)\frac{\partial^{2}T}{\partial {\bar{y}}^{2}}+\tau_{1}\left[D_{B}\frac{\partial C}{\partial \bar{y}}\frac{\partial T}{\partial \bar{y}}+\frac{D_{T}}{T_{\infty }}{\left(\frac{\partial T}{\partial \bar{y}}\right)}^{2}\right],$$

(4) $$\frac{\partial C}{\partial t}+u\frac{\partial C}{\partial \bar{x}}+v\frac{\partial C}{\partial \bar{y}}=D_{B}\frac{\partial^{2}C}{\partial {\bar{y}}^{2}}+\frac{D_{T}}{T_{\infty }}\frac{\partial^{2}T}{\partial {\bar{y}}^{2}}-k_{c}\left(C-C_{\infty }\right),$$

(5) $$\frac{\partial n}{\partial t}+u\frac{\partial n}{\partial \bar{x}}+v\frac{\partial n}{\partial \bar{y}}+\frac{b_{1}W_{c}}{\left(C_{w}-C_{\infty }\right)}\left[\frac{\partial }{\partial y}\left(n\frac{\partial C}{\partial \bar{y}}\right)\right]=D_{m}\left(\frac{\partial^{2}n}{\partial {\bar{y}}^{2}}\right),$$

We assign following boundary conditions for the current flow problem

(6) $$u=u_{\omega }=b\bar{x}\sin\;\omega t,\;\;\;v=0,\;\;T=T_{w},\;\;C=C_{w}\text{,}n=n_{w}\text{ at}\;\;\;\bar{y}=0,\;\;t>0,$$

(7) $$u\to 0,\;\;T\to T_{\infty }\text{, }C\to C_{\infty }\text{,}n\to n_{\infty }\text{ at}\;\;\;\;\bar{y}\to \infty .$$

The initial conditions are

(8) u=0,v=0    t=0.

where Γ is time constant, $\rho_{f}$ represents the fluid density, $k^{*}$ denotes the permeability parameter, φ is porous medium, $\sigma^{*}$ electrical conductivity, $\rho_{p}$ density of nanoparticles, $\rho_{m}$ microorganisms particles, $\beta^{*}$ is volume expansion coefficient, g the gravity, T is temperature, $\sigma_{m}$ determine the Stefan Boltzmann constant and $k^{*}$ is the absorption constant, C is concentration, $D_{B}$ denotes coefficient of Brownian diffusion, $D_{T}$ reports the thermophoretic diffusion constant, $\tau_{1}={\left(\rho c\right)}_{p}/{\left(\rho c\right)}_{f}$ signify the nanoparticles heat capacitance and fluid particles heat capacitance ratio, $D_{m}$ relates the microorganisms density, n stands for gyrotactic microorganism density, $W_{c}$ is swimming cell speed while $b_{1}$ is the chemotaxis constant.

Before compute the desired solution of the flow problem, first we transform the flow problem in dimensionless form by initiating following variables [17].

(9) $$u=b\bar{x}f_{y}\left(y,\;\tau \right),\;\;v=-\sqrt{\nu b}f\left(y,\;\tau \right)\;,\;\;\;y=\sqrt{\frac{b}{\nu }}\bar{y},\;\;\tau =t\omega ,$$

(10) $$\theta \left(y,\;\tau \right)=\frac{T-T_{\infty }}{T_{w}-T_{\infty }},\phi \left(y,\;\tau \right)=\frac{C-C_{\infty }}{C_{w}-C_{\infty }},\chi \left(y,\;\tau \right)=\frac{n-n_{\infty }}{n_{w}-n_{\infty }},$$

The substitution of above variables in Eqs. (2), (3), (4), and (5), following dimensionless forms are recovered

(11) $$f_{yyy}-Sf_{y\tau }-f_{y}^{2}+ff_{yy}-\beta f_{y}+Wef_{yy}f_{yyy}+\lambda \left(\theta -N_{r}\varphi -R_{b}\chi \right)=0,$$

(12) $$\left[1+\frac{4}{3}Rd{\left\{1+\left(\theta_{w}-1\right)\theta \right\}}^{3}\right]\theta_{yy}+Rd\left[3\left(\theta_{w}-1\right)\theta_{y}^{2}\right]{\left[1+\left(\theta_{w}-1\right)\theta \right]}^{2}+\mathrm{Pr}\left(f\theta_{y}-S\theta_{\tau }+\delta \theta \right)=0,$$

(13) $$\varphi_{yy}-S\left(Sc\right)\varphi_{\tau }+Scf\varphi_{y}+\frac{Nt}{Nb}\theta_{yy}-KrSc\varphi =0,$$

(14) $$\chi_{yy}-SLb\chi_{\tau }+Lb\chi_{y}-Pe\left[\chi_{y}\varphi_{y}+\varphi_{yy}\left(\chi +\sigma \right)\right]=0,$$

The governing boundary conditions are

(15) $$f_{y}\left(0,\;\tau \right)=\sin\;\tau ,\;\;f\left(0,\;\tau \right)=0,\;\theta \left(0,\tau \right)=1,\varphi \left(0,\tau \right)=1,\chi \left(0,\tau \right)=1,$$

(16) $$f_{y}\left(\infty ,\;\tau \right)\to 0,\;\;\;\;\theta \left(\infty ,\tau \right)\to 0,\varphi \left(\infty ,\tau \right)\to 0,\chi \left(\infty ,\tau \right)\to 0,$$

where $We=2\Gamma \bar{x}\sqrt{2b^{3}/\nu }$is the local Williamson parameter, S=ω/bis the ratio of oscillation frequency to stretching rate, $\beta =\sigma^{*}B_{0}^{2}/\rho_{f}b+\nu \varphi /k^{*}b$ the combined Hartmann number and porosity parameter (combined parameter), $\lambda =\beta^{*}g\left(1-C_{\infty }\right)\left(T_{w}-T_{\infty }\right)/a^{2}x$ the mixed convection parameter, $Rb=\gamma^{*}\left(n_{w}-n_{\infty }\right)\left(\rho_{m}-\rho_{f}\right)/\beta^{*}\rho_{f}\left(1-C_{\infty }\right)\left(T_{w}-T_{\infty }\right)$ bioconvected Rayleigh number, $\mathrm{Pr}=\nu /\alpha_{f}$ the Prandtl number, $Rd=4\sigma^{*}{T_{\infty }}^{3}/3kk^{*}$is the radiation parameter, $\theta_{w}=T_{w}/T_{\infty }$ is the surface heating parameter, $Nr=\left(\rho_{p}-\rho_{f}\right)\left(C_{w}-C_{\infty }\right)/\beta^{*}\rho_{f}\left(1-C_{\infty }\right)T_{\infty }\beta$ buoyancy ratio parameter, $Nt=\tau_{1}D_{T}\left(T_{w}-T_{\infty }\right)/T_{\infty }\nu$the thermophoresis parameter, $Sc=\nu /D_{B}$ the Schmidt number,$Nb=\tau_{1}D_{B}\left(C_{f}-C_{\infty }\right)/\nu$ the Brownian motion parameter, $Lb=\nu /D_{m}$ the bioconvected Lewis number, $Kr=k_{a}/b$ is the chemical reaction parameter,$Pe=b_{1}W_{c}/D_{m}$ the Peclet number and $\sigma =n_{\infty }/n_{w}-n_{0}$ is the microorganisms concentration difference parameter.

We expressed the physical quantities namely local Nusselt number, local Sherwood number and local motile number in following forms:

(17) $$\begin{array}{l} Nu_{x}=\frac{\bar{x}q_{s}}{k\left(T_{w}-T_{\infty }\right)},\;Sh_{x}=\frac{\bar{x}j_{s}}{D_{B}\left(C_{w}-C_{\infty }\right)},\;Nn_{x}=\frac{\bar{x}g_{s}}{D_{m}\left(n_{w}-n_{\infty }\right)},\\ q_{s}=-k{\left(\frac{\partial T}{\partial \bar{y}}\right)}_{\bar{y}=0},j_{s}=-D_{B}{\left(\frac{\partial C}{\partial \bar{y}}\right)}_{\bar{y}=0},g_{s}=-D_{m}{\left(\frac{\partial n}{\partial \bar{y}}\right)}_{\bar{y}=0}, \end{array}$$

After inserting the dimensionless quantities in above expression, we have

(18) NuxRex−0.5=−(1+43Rdθw3)θy(0,τ),ShxRex−0.5=−φy(0,τ),NnxRex−0.5=−χy(0,τ),

where $Nu_{x}$ local Nusselt number, $Sh_{x}$ is local Sherwood and $Nn_{x}$ local motile organisms density number.

## 3 Homotopy analysis method

In various engineering, technological and industrial systems, the resulted differential equations are of highly nonlinear in nature which always poses a challenge for mathematicians and engineers. Such problems are often treated numerically or analytically. Beyond the analytical techniques, homotopy analysis method is one which successfully computes the desired series solution. One of the astonishing aspect of this method is it does not restrict the condition of large or small parameter. The region of convergence associated with this technique can be addressed more conveniently as compared to other techniques. It offers great freedom to develop the desired base functions to compute the solution. The pioneer work on this method was initiated by Liao [28] and later on many investigators use this technique for various nonlinear problems [29, 30, 31, 32]. Following initial guesses are suggested to start the simulations

(19) $$f_{0}\left(y,\tau \right)=\sin\;\tau \left(1-e^{-y}\right),\;\;\theta_{0}\left(y\right)=e^{-y},\;\;\varphi_{0}\left(y\right)=e^{-y},\chi_{0}\left(y\right)=e^{-y}.$$

Let us assert the auxiliary linear operators to start the analytical simulations

(20) $${£}_{f}=\frac{\partial^{3}}{\partial y^{3}}-\frac{\partial }{\partial y},\;\;\;{£}_{\theta }=\frac{\partial^{2}}{\partial y^{2}}-1,\;{£}_{\varphi }=\frac{\partial^{2}}{\partial y^{2}}-1,\;{£}_{\chi }=\frac{\partial^{2}}{\partial y^{2}}-1,$$

satisfying

(21) $${£}_{f}\left[c_{1}+c_{2}e^{y}+c_{3}e^{-y}\right]=0,$$

(22) $${£}_{\theta }\left[c_{4}e^{y}+c_{5}e^{-y}\right]=0,$$

(23) $${£}_{\varphi }\left[c_{6}e^{y}+c_{7}e^{-y}\right]=0,$$

(24) $${£}_{\chi }\left[c_{8}e^{y}+c_{9}e^{-y}\right]=0,$$

where $c_{i}\left(i=1,\;2,...,9\right)$ represents arbitrary constants.

### 3.1 Convergence analysis

**Figure 1:**
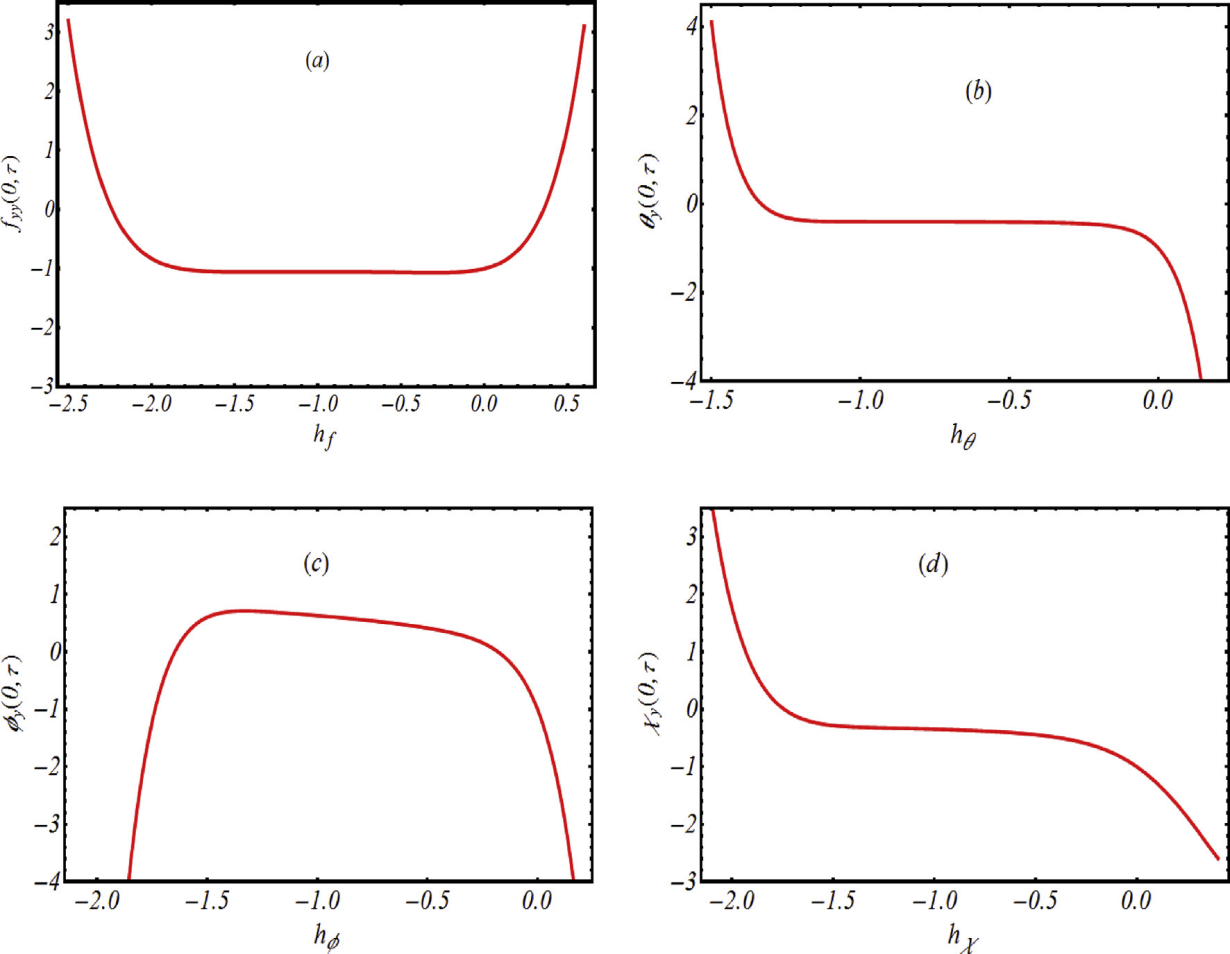
h−curves for (a) velocity profile, (b) temperature profile, (c) concentration profile and (d) motile micro-organisms profile.

The simulations based on HAM method results a series solution which involves the auxiliary constants $h_{f}$, $h_{\theta }$, $h_{\varphi }$ and $h_{\chi },$ for which suitable selection of these auxiliary constants are quite necessitated. For this purpose, h−curves for velocity, temperature, concentration and motile micro-organisms profiles are presented for specified values of emerging parameters in Figure 1(a-d). It is pointed out that more convenient values for the given solution are selected from $-2.1\leq h_{f}\leq 0,$ $-1.8\leq h_{\theta }\leq 0.1,$ $-1.7\leq h_{\varphi }\leq 0.2,-1.7\leq h_{\chi }\leq 0.2.$

### 3.2 Validation of results

Before analyze the graphical results, first we verify our solution by comparing it with already reported data. The obtained results are compared as a limiting case with exact solution, suggested by Turkyilmazoglu [33] and Hayat et al. [34] in Table 1. Table shows that our results meet good agreement with these results. Present numerical computations are also compared with Zheng et al. [35] and Abbas et al. [36] in Table 2 for various values of τ. Again an excellent agreement of our results has been noted with these studies.

**Table 1:** Numerical values of f''(0,τ) for linear stretching with We=S=λ=Nr=Rb=0 and τ=π/2.

β	Turkyilmazoglu [33]	Hayat et al. [34]	Present results
0	-1.000000	-1.000000	-1.000000
0.5	-1.224744	-1.224747	-1.224747
1	-1.414213	-1.414217	-1.414217
1.5	-1.581138	-1.581147	-1.581147
2.0	-1.732050	-1.732057	-1.732057

**Table 2:** Comparison of f''(0,τ) with [35, 36] when S=1,β=12,λ=0,Nr=0 and Rb=0.

τ	Zheng et al. [35]	Abbas et al. [36]	Present results
τ=1.5π	11.678656	11.678656	11.678656
τ=5.5π	11.678706	11.678707	11.678706
τ=9.5π	11.678656	11.678656	11.678656

## 4 Discussion

After computing the desired solution, now we examine the rheological behavior of various fluid parameters on velocity profile $f_{y}$, temperature profile θ, nanoparticles volume fraction φ and motile microorganism profile χ. It is remarked that while varying each flow parameter, the remaining parameters have assigned some constant values to like We=0.5,β=0.5, λ=0.2, Nr=0.1, Rb=0.4, Rd=0.2, Nt=0.3, Nb=0.3, Pr=0.71,Sc=0.4, Kr=0.2, Pe=0.5, Lb=0.5. The detailed physical significance of each parameter is discussed in this section.

### 4.1 Velocity profile

**Figure 2:**
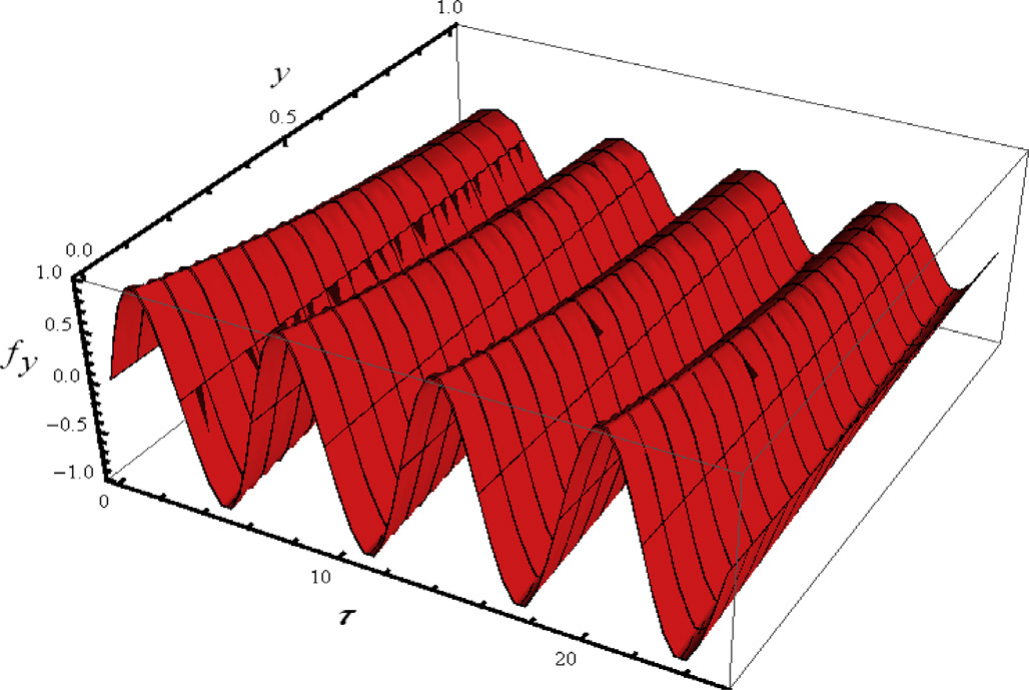
Flow phenomenon of ξ and τ verses $f_{\xi }.$

**Figure 3:**
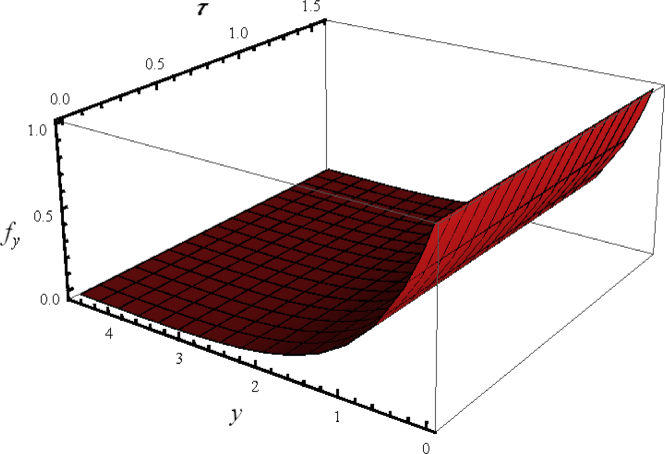
Flow phenomenon of ξ and τ verses $f_{\xi }.$

Since flow is time depend so first we examine the three dimensional (3D) illustration of velocity profile $f_{y}$ with y and time τ. For this purpose, Figure 2 is plotted for some fixed values of emerging parameters. The velocity distribution oscillates periodically with time near the surface. Further, a phase shift in the distribution of velocity has also been captured far away from the accelerated surface. Figure 3 portrayed the 3D variation in velocity distribution when all the flow parameters have assigned some constant values. It is noted that velocity distribution gradually varied along y− direction without oscillation.

### 4.2 Temperature profile

**Figure 4:**
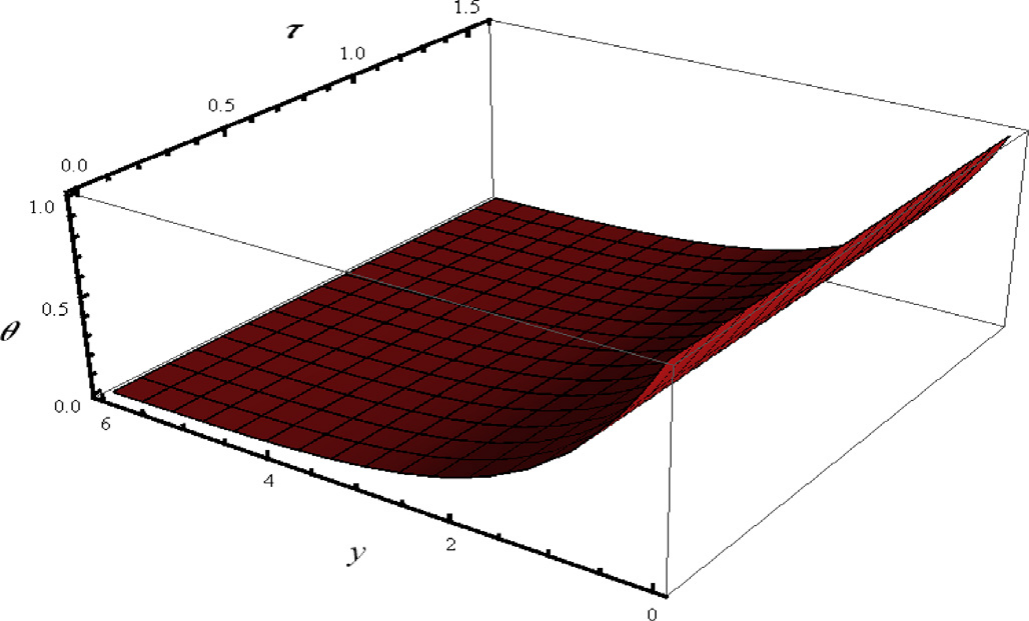
Flow phenomenon of ξ and τ verses θ.

In current analysis we have examined bioconvection of non-Newtonian nanofluid in presence of nonlinear thermal radiation. Now we determined the graphical analysis for temperature distribution θ. On this end, 3D visualization of temperature θ with y and τ is plotted in Figure 4. The temperature distribution varied linearly without any oscillation.

### 4.3 Concentration profile

**Figure 5:**
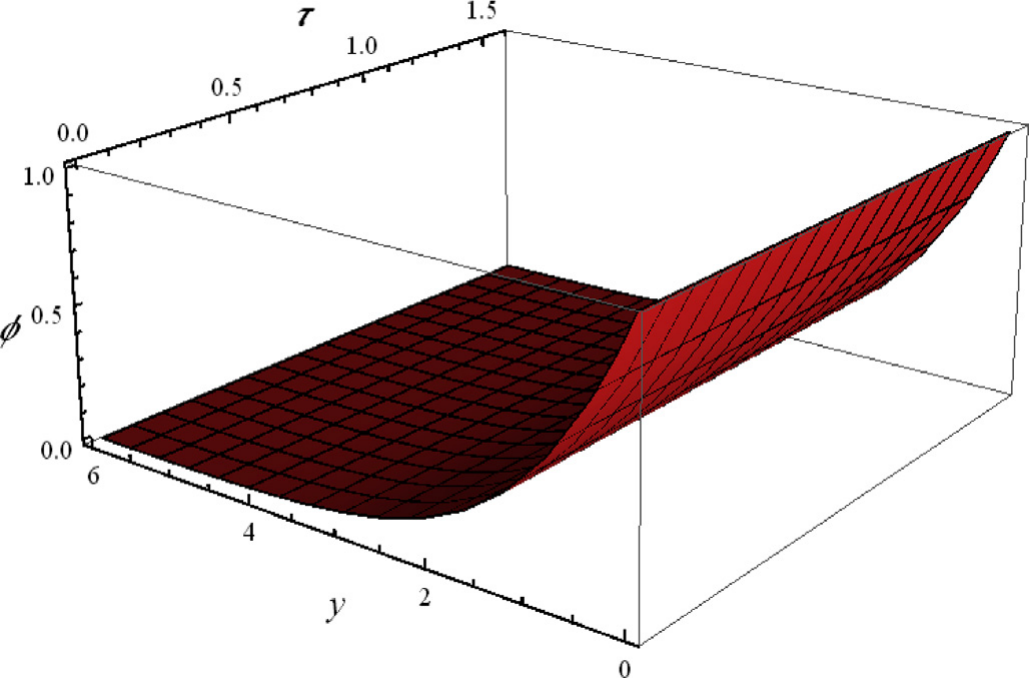
Flow phenomenon of ξ and τ verses φ.

The role of Williamson parameter We, Schmidt number Sc, mixed convection λ, buoyancy ratio Nr, bioconvected Rayleigh number Rb, thermophoresis Nt and Brownian motion Nb on nanoparticles volume fraction φ is visualize in Figures 4 and 5. Again, the graphical analysis has been performed with 3D visualization. It is noted that nanoparticles volume fraction φ linearly varied against y. It is noted that nanoparticles volume fraction φ is not truncated with time efficiently.

### 4.4 Motile microorganism profile

**Figure 6:**
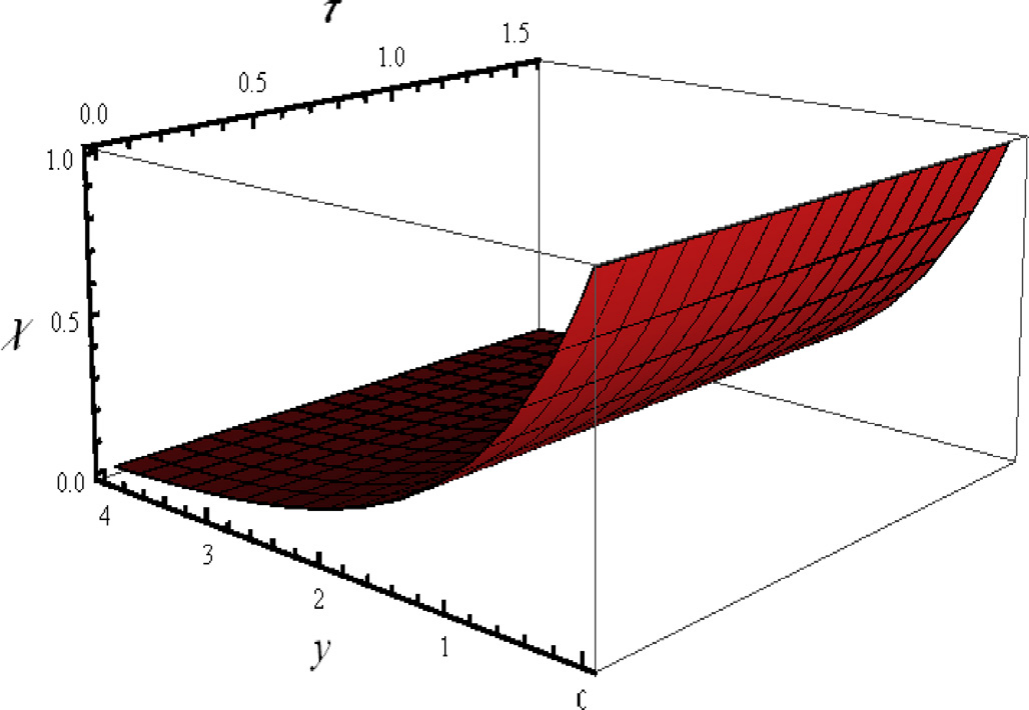
Flow phenomenon of ξ and τ verses χ.

Figure 6 concentrates the 3D illustration of microorganisms distribution χ when all the parameters have assigned fixed numerical values. From this figure, we observed that microorganisms distribution χ is varied linearly again.

### 4.5 Physical quantities

The variation in the local Nusselt number, local Sherwood number and motile density number for flow parameters are portrayed in Table 3. An increasing variation in these physical quantites is taken out for Prandtl number while decreasing behavior has been observed for Williamson parameter. Similarly, these quantities get lower values of mixed convection parameter and Rayleigh constant.

**Table 3:** The variation in $-\theta_{y}\left(0,\tau \right),$$-\varphi_{y}\left(0,\tau \right)$ and $-\xi_{y}\left(0,\tau \right)$ for various flow parameters.

We	Pr	λ	Nt	Nb	Nr	Rb	$-\theta_{y}\left(0,\tau \right)$	$-\varphi_{y}\left(0,\tau \right)$	$-\xi_{y}\left(0,\tau \right)$
0.0	0.7	0.2	0.2	0.2	0.2	0.2	0.681088	0.52956	0.809282
0.5							0.691838	0.540209	0.819285
1.5							0.700140	0.551419	0.849292
0.2	0.1						0.725145	0.536187	0.880311
	0.7						0.807551	0.748766	0.923611
	1.0						0.841138	0.751677	0.944499
	0.7	0.0					0.822811	0.748896	0.936444
		0.5					0.822723	0.748601	0.934341
		1.5					0.822412	0.748313	0.931122
		0.2	0.0				0.741395	0.754331	0.880266
			0.8				0.63338	0.409362	0.878720
			1.2				0.606906	0.29585	0.847932
			0.2	0.5			0.701822	0.550416	1.01144
				0.6			0.652736	0.677405	1.00142
				1.0			0.634843	0.717644	0.99951
				0.2	0.0		0.702159	0.570572	1.08437
					0.5		0.681844	0.560216	1.08422
					1.0		0.661326	0.559771	1.08404
					0.2	0.0	0.702333	0.575636	1.08437
						0.5	0.700844	0.571216	1.08436
						1.0	0.690326	0.566671	1.08435

## 5 Conclusions

This study reports the thermophoresis and Brownian effects in flow of Williamson nanoparticles gyrotactic microorganisms. The flow has been assumed over an accelerated surface. As novelty, nonlinear thermal radiation effects are also utilized. First physical phenomenon is formulated by using boundary layer approximations. The series solution is acquired by using HAM. The solution with excellent accuracy has been obtained and results are compared with already reported continuations. A detailed graphical analysis has been performed by illustrating 3D visualization. It is found that velocity distribution accelerate periodically for flow parameters. The 3D simulations for temperature, concentration and microorganisms distribution does not contains any periodic oscillation. The detected observation can involve theoretical significance in various engineering processes, bio-fuel cells, solar energy system and enhancement of extrusion systems.

## Declarations

### Author contribution statement

Kamel Al-Khaled: Conceived and designed the experiments; Analyzed and interpreted the data.

Sami Ullah Khan: Contributed reagents, materials, analysis tools or data; Wrote the paper.

Ilyas Khan: Performed the experiments; Analyzed and interpreted the data.

### Funding statement

This research did not receive any specific grant from funding agencies in the public, commercial, or not-for-profit sectors.

### Competing interest statement

The authors declare no conflict of interest.

### Additional information

No additional information is available for this paper.
